# Infection of Two Heterologous Mycoviruses Reduces the Virulence of *Valsa mali*, a Fungal Agent of Apple Valsa Canker Disease

**DOI:** 10.3389/fmicb.2021.659210

**Published:** 2021-05-25

**Authors:** Shian Yang, Ruoyin Dai, Lakha Salaipeth, Lili Huang, Jie Liu, Ida Bagus Andika, Liying Sun

**Affiliations:** ^1^State Key Laboratory of Crop Stress Biology for Arid Areas, College of Plant Protection, Northwest A&F University, Xianyang, China; ^2^School of Bioresources and Technology, King Mongkut’s University of Technology Thonburi, Bangkok, Thailand; ^3^College of Plant Health and Medicine, Qingdao Agricultural University, Qingdao, China

**Keywords:** mycovirus, hypovirus, mycoreovirus, *Valsa mali*, hypovirulence, RNA silencing

## Abstract

Mycovirus infection has been widely shown to attenuate the virulence of phytopathogenic fungi. *Valsa mali* is an agriculturally important fungus that causes Valsa canker disease in apple trees. In this study, two unrelated mycoviruses [*Cryphonectria hypovirus* 1 (CHV1, genus *Hypovirus*, and single-stranded RNA) and Mycoreovirus 1 (MyRV1, genus *Mycoreovirus*, double-stranded RNA)] that originated from *Cryphonectria parasitica* (chestnut blight fungus) were singly or doubly introduced into *V. mali* via protoplast fusion. CHV1 and MyRV1 stably infected *V. mali* and caused a reduction in fungal vegetative growth and virulence. Co-infection of both viruses further reduced the virulence of *V. mali* but compromised the stability of CHV1 infection and horizontal transmission through hyphal anastomosis. Infections of MyRV1 and, to a lesser extent, CHV1 up-regulated the transcript expression of RNA silencing-related genes in *V. mali*. The accumulation of CHV1 (but not MyRV1) was elevated by the knockdown of *dcl2*, a key gene of the RNA silencing pathway. Similarly, the accumulation of CHV1 and the efficiency of the horizontal transmission of CHV1 during co-infection was restored by the knockdown of *dcl2*. Thus, CHV1 and MyRV1 are potential biological control agents for apple Valsa canker disease, but co-infection of both viruses has a negative effect on CHV1 infection in *V. mali* due to the activation of antiviral RNA silencing by MyRV1 infection.

## Introduction

Apple Valsa canker disease is a destructive plant disease that affects apple trees particularly in East Asian countries (Lee et al., 2006; Abe et al., 2007). The elongated cankers occur on the branches and the trunk, leading to the death of the tree and failure of the entire orchard (Chen et al., 1987). This plant disease is widespread in many apple-producing regions in China and results in significant yield losses (Wang et al., 2011; Li et al., 2013). Apple Valsa canker is caused by the plant pathogenic ascomycete fungus, interchangeably referred to as *Valsa mali* or *Valsa ceratosperma* due to synonymization of *V. mali* that was initially identified as a new species for the causative pathogen of apple Valsa canker, to *V. ceratosperma*. Although *V. ceratosperma* was later found to be a heterogeneous species complex, further analyses revealed that apple strains of *V. mali* and *V. ceratosperma* from East Asia represent the same fungal species (Wang et al., 2011, 2014, 2020). Apple Valsa canker is difficult to control using chemical treatments even though many types of fungicide have been tested (Wang et al., 2009). Currently, strategies for controlling the disease include strict cultivation management and scraping away of lesions before the application of fungicides. However, highly effective prevention and control measures for this disease are not available (Keqiang et al., 2009).

Research in the field of plant pathology has been aiming to the development of sustainable control methods for fungal diseases that have minimal negative effects on the environment and human health (Raymaekers et al., 2020). In this regard, the biological control method which utilizes mycoviruses (fungal viruses) as control agents (virocontrol) is a promising method for the protection of plants against phytopathogenic fungi (Xie and Jiang, 2014; García-Pedrajas et al., 2019). Mycoviruses are widespread among fungal groups (Ghabrial and Suzuki, 2009). Several species of mycoviruses can reduce the virulence of their host, however, most do not affect the host (Hillman et al., 2018). The mycovirus *Cryphonectria hypovirus* 1 (CHV1) has been successfully used in Europe as a biological control agent to manage chestnut blight caused by the plant pathogenic fungus *Cryphonectria parasitica* (Rigling and Prospero, 2018). CHV1 infection is associated with attenuated virulence, reduced pigmentation, suppressed asexual sporulation, and altered expression of certain genes of its host (Dawe and Nuss, 2001). CHV1 is a non-segmented, positive-sense, single-stranded RNA virus belonging to the genus *Hypovirus* of the family *Hypoviridae*. Its genome (12.7 kilobases) contains two open reading frames (ORFs), ORF A and ORF B. These ORFs encode multifunctional polyproteins such as the papain-like proteases, p29 and p48, which are responsible for proteolytic cleavage in the generation of functional viral proteins (Shapira et al., 1991b). The p29 encoded in CHV1 ORF A is a multifunctional protein which plays a role in the suppression of host pigmentation, sporulation, and RNA silencing (Craven et al., 1993; Sun et al., 2009; Chiba and Suzuki, 2015).

Besides CHV1, Mycoreovirus 1 (MyRV1), isolated from *C. parasitica* in the United States, is a potential biological control agent. MyRV1 is a member of the genus *Mycoreovirus* within the family *Reoviridae*. Its genome contains 11 segments of double-stranded RNA (dsRNA; S1 to S11) ranging from 4127 to 732 base pairs (bp) in length (Hillman et al., 2004). MyRV1 markedly reduces the virulence of its host, but it shows minimal effects on pigmentation and asexual sporulation (Hillman et al., 2004). Mixed infection of CHV1 and MyRV1 in *C. parasitica* presents a one-way synergism in which CHV1 enhances the replication and vertical transmission of MyRV1 through asexual spores (Sun et al., 2006).

A previous study showed that introduction of CHV1 into *V. ceratosperma* via biolistic delivery of infectious cDNA clone results in colony morphology changes and reduced virulence (Sasaki et al., 2002). Another study demonstrated that infection of Rosellinia necatrix Mycoreovirus 3, a mycoreovirus originated from the white root rot fungus *Rosellinia necatrix* in *V. ceratosperma* causes reduction in fungal virulence (Kanematsu et al., 2010). A more recent study identified a new hypovirus naturally infecting *V. ceratosperma*, but the infection of this virus is not associated with reduction of fungal virulence (Yaegashi et al., 2012).

RNA silencing is a sequence-specific gene downregulation that is also important as an antiviral defense mechanism in eukaryotes including fungi (Aliyari and Ding, 2009; Chang et al., 2012). In antiviral RNA silencing, viral-derived dsRNAs are cleaved by a Dicer-like protein (DCL) to generate small interfering RNAs that guide an Argonaute (AGO)-containing RNA silencing-induced complex for sequence-specific degradation of a viral RNA target (Ding, 2010). Ascomycete fungi are known to encode two DCLs and a varying number of AGOs, according to the species of fungus (Nakayashiki et al., 2006). The *V. mali* genome contains two *dcl* genes (*dcl1* and *dcl2*) and three Argonaute-like (*agl*) genes (*agl1*, *agl2*, and *agl3*; Feng et al., 2017a,b) but their roles in antiviral defense responses have not been demonstrated.

In this study, we examined the effects of single and double infection of CHV1 and MyRV1 on vegetative growth and the virulence of *V. mali*. Our results suggest that CHV1 and MyRV1 are potential biological control agents for *V. mali* but co-infection of both viruses compromised the stability of CHV1 infection. Furthermore, we examined the interaction of CHV1 and MyRV1 with antiviral RNA silencing in *V. mali* in the context of single and double infection.

## Materials and Methods

### Fungal Strains and Viruses

The *C. parasitica* strains EP713 (Nuss et al., 2005) and 9B21 (Hillman et al., 2004), naturally infected with CHV1 and MyRV1, respectively, were provided by Dr. Nobuhiro Suzuki from Okayama University in Japan. The wild-type and *dcl2* knockout mutants of *V. mali* strains were described previously (Feng et al., 2017a).

All fungal strains were grown for 3 to 5 days on potato dextrose agar (PDA, Difco) under benchtop conditions at 24∼26°C for morphological observation or in potato dextrose broth (PDB, Difco) when mycelia were used for the preparation of protoplasts. For maintenance, fungal strains were cultured on regeneration plates (Churchill et al., 1990) and stored at 4°C until further use. To observe the formation of asexual fruiting bodies, the fungal strains were continually cultured on PDA for 4 to 5 additional weeks.

### Protoplast Preparation

The protoplasts of all fungal strains were prepared individually using a method described previously (Eusebio-Cope et al., 2009) with a slight modification. The fungal strains grown on PDA medium were cut into small pieces, cultured in 20 ml PDB (50 ml flask) and incubated in the dark at 25°C for 3 days without shaking. The young mycelia were harvested, washed with 0.6 M MgSO_4_ and suspended in an enzymatic solution containing 3∼6 mg lysine enzyme, 10∼15 mg β-glucuronidase and 6∼12 mg bovine serum albumin in 10 ml of 1.2 M MgSO_4_. This was followed by incubation at 27°C for 3∼4 h with gentle shaking. The protoplast suspensions were slowly poured into a new 50 ml plastic tube. A 1.25 volume of trapping buffer (0.4 M sorbitol in 100 mM Tris–HCl, pH 7.0) was overlaid onto the protoplast suspensions and centrifuged at 3,500 rpm for 20 min at 4°C to concentrate the protoplasts at the interface. The protoplasts were diluted in 2 volumes of 1 M sorbitol, centrifuged at 3,500 rpm for 20 min at 4°C and washed again with 10 ml of STC (1 M sorbitol, 100 mM CaCl_2_, 100 mM Tris–HCl, and pH 8.0). Finally, the protoplasts were suspended in a small volume of STC to obtain approximately 1 × 10^7^ protoplasts per ml. This suspension was immediately used or stored at –80°C until further use.

### Protoplast Fusion

To introduce CHV1 and MyRV1 into *V. mali*, the protoplasts prepared from *C. parasitica* strains EP713 (infected with CHV1) or 9B21 (infected with MyRV1) were fused with *V. mali* protoplasts using polyethylene glycol (PEG). To obtain doubly infected strain, *V. mali* protoplasts were simultaneously fused with the protoplasts prepared from both *C. parasitica* strains EP713 and 9B21. *V. mali* protoplasts (100 μl 1 × 10^7^ of protoplasts/ml) and *C. parasitica* protoplasts (100∼200 μl of 1 × 10^6^ protoplasts/ml) were mixed gently and placed on ice for 30 min. After adding 500 μl of PTC solution (40% PEG 4000, 100 mM Tris–HCl pH 8.0, and 100 mM CaCl_2_) to each protoplast suspension, the mixtures were combined gently and incubated at room temperature for 20 min. The protoplast mixtures were then centrifuged at 3,500 rpm for 5 min at 4°C, resuspended with 100 ml of 1 M sorbitol, divided into 10 aliquot parts and each placed in the center of a petri dish, and then 20∼25 ml of YCDA (0.1% yeast extract, 0.1% casein hydrolysate, 0.5% glucose, and 1.5% agar) was added. The Petri dishes were left on the benchtop for 5 to 7 days and a small piece of hypha from the edges of the colonies was subsequently transferred to new PDA plates and examined for the *V. mali* phenotype or prepared for dsRNA extraction. To ensure the homogeneity of *V. mali* isolates obtained from protoplast fusion, the fungal isolates were cultured on PDA plates from single hypha of fungal colonies that grew after regeneration of protoplasts. Basically, *V. mali* can be isolated due to the faster growth of *V. mali* on YCDA or PDA medium than that of *C. parasitica*. Furthermore, the purity of *V. mali* isolates obtained from the protoplast fusion is confirmed by observation of fungal colony morphology and sequencing of ribosomal-DNA intergenic spacer regions.

### Horizontal Transmission of Viruses

The horizontal transmission of viruses through hyphal anastomosis was performed as described previously (Chiba et al., 2013). The virus-infected donor strain was cultured with a virus-free recipient strain at a distance of 1 cm on a PDA plate and incubated at 24∼26°C. After 5 or 7 days of hyphal contact, mycelial plugs were taken from three different positions (near, middle, and far) on the recipient side and cultured on PDA plates layered with cellophane for 4 days before RNA extraction for viral dsRNA analysis or RT-PCR detection.

### Phenotypic and Virulence Assays

The growth of fungal colonies on a PDA plate (maintained on a benchtop at 24∼26°C for 4 days) was measured based on the colony area of three replicates. The morphology of the fungi was analyzed and photographed. Virulence has been measured in apples according to the area of the lesions induced by fungal growth (Hillman et al., 1990). Apples (cv. Gala) were purchased from a supermarket and 2-year-old apple twigs were obtained from apple trees planted at an experimental station at Northwest A&F University, China. The fruits and branches were washed with tap water, the surface was wiped with 75% ethanol and they were then rinsed several times with sterile distilled water. Small holes of 6 mm in diameter were created on the surface of the fruits and branches, after which an agar plug from the growing margin of a 4-day-old colony was placed directly on each hole and wrapped with parafilm. The apples were placed in a container with wet tissue paper, and the ends of each twig were covered with wet tissue paper. All samples were maintained at 25°C for 5 or 10 days. The parafilm wraps were removed 2 days after inoculation. After 5 or 10 days of inoculation, lesion sizes were measured as indicators of disease development. Each sample contained five inoculation points, and five apples, and five twigs were used for each strain. Virulence assays were repeated three times independently.

### dsRNA Isolation

A rapid, small-scale extraction of dsRNA was performed. Fungal mycelia grown for 4 days were collected from PDA plates layered with cellophane or PDB cultures, then ground into a powder with liquid nitrogen and homogenized with 1 ml of EBA buffer (50 mM Tris–HCl pH 8.5, 50 mM EDTA, 3% SDS, 1% PVPP, and 1% DTT). The suspensions were centrifuged at 4°C for 15 min at 12,000 rpm. The supernatant (600 μl) was mixed with 600 μl of STE (10 mM Tris–HCl pH 8.0, 1 mM EDTA, and 150 mM NaCl), ethanol (up to 16% volume) and a small amount of CC41 cellulose in preparation for column chromatography analysis of dsRNAs. After 40∼60 min of continuous agitation at room temperature, each tube was washed three times with STE-EtOH (16% EtOH; v/v), with vortexing and centrifugation between washes. The dsRNAs were eluted from the dried CC41 by adding 700 μl of STE buffer and centrifuging at 12,000 rpm for 2 min. The eluate was collected and mixed with an equal volume of isopropanol, incubated for 10 min at room temperature and centrifuged at 4°C for 30 min at 12,000 rpm. The dsRNA pellet was washed with 70% ethanol, air-dried at room temperature and dissolved in 30∼50 μl of RNase-free water. Each sample was subjected to 1.4% agarose gel electrophoresis in 1X TBE buffer.

### Total RNA Extraction and Viral dsRNA Quantification

Total RNA was prepared from *V. mali* mycelia cultured in PDA with cellophane for 3 days, as described previously (Sun et al., 2006). The RNA concentration was adjusted to 2 μg/μl using a Nano Photometer (N50 Touch) for use in agarose gel electrophoresis. Viral genomic dsRNA was quantified using densitometry, as described previously (Suzuki et al., 2003). Total RNA samples were analyzed by electrophoresis using a 1.4% agarose gel in a 1X TAE (40 mM Tris/acetate pH 7.8, 1 mM EDTA) buffer system and stained with ethidium bromide. The RNA bands were visualized using a UV lamp under a transilluminator and were photographed digitally at various exposures. The RNA bands were analyzed and quantified using Image J Macro software. Relative amounts of MyRV1 genomic RNA were quantified by measuring the amount of S1, S2, and S3 RNA segments normalized to the amount of host fungal 18S rRNA. A similar method was used to quantify CHV1 genomic RNA accumulation.

### RT-PCR and Quantitative PCR Analyses

The single stranded RNAs (ssRNAs) were extracted from fungal mycelia (3 days old) following the procedure described previously (Sun et al., 2006). First-strand cDNA was synthesized with ReverTra Ace reverse transcriptase (Toyobo) using 0.2 μg of ssRNAs as a template (according to the manufacturer’s instructions) and then used for PCR amplification using 2-mixture DNA polymerase (Kangwei) and primers specific for CHV1 p29. Quantitative PCR was performed using a CRX96TM Real-Time PCR Detection System (Bio-Rad) via a Kapa SYBR Fast ABI Prism qPCR mix kit (Kapa Biosystems). The 18S RNA of *V. mali* was used as an internal control. Three biological replicates were used for each sample and the experiments were repeated three times independently. The primers used in this study are listed in Supplementary Table 1.

### Statistic Analysis

One-way ANOVA analysis was performed in Microsoft Excel with *post hoc t*-tests.

## Results and Discussion

### CHV1 and MyRV1 Can Infect *V. mali*

**FIGURE 1:**
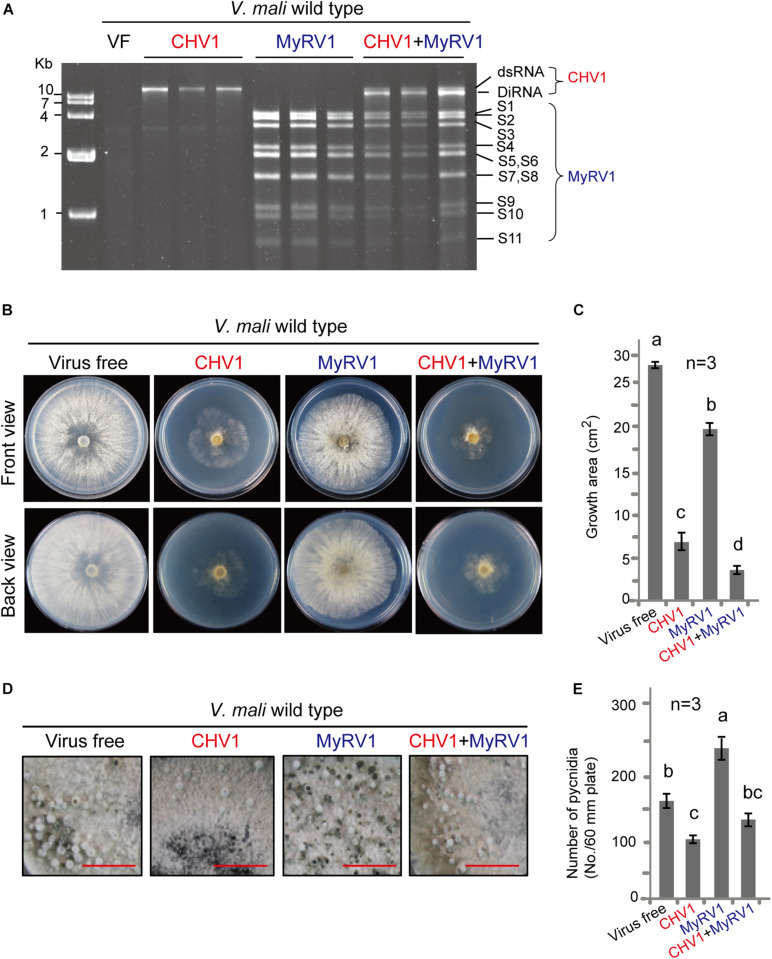
Infection of CHV1 and MyRV1 in *V. mali*. **(A)** Agarose gel electrophoresis of dsRNAs extracted from *V. mali* strains singly or doubly infected with CHV1 and MyRV1. The dsRNA samples were analyzed via electrophoresis in an agarose gel stained with ethidium bromide. The CHV1 dsRNA bands that migrated slightly faster than the wild-type CHV1 are defective interference RNA (DI-RNA). **(B)** Phenotypic growth on PDA medium of representative *V. mali* strains singly or doubly infected with CHV1 and MyRV1. The colonies were grown on PDA for 4 days and then photographed. **(C)** The growth areas of fungal strains described in **(B)**. The data are the means ± SD (*n* = 3). The different letters indicate a significant difference at *p* < 0.01 (one-way ANOVA). **(D)** Representative images showing the formation of asexual fruiting bodies (pycnidia) of the fungal strains described in **(B)**. The fungi were cultured on PDA medium for 4–5 weeks until the pycnidia were produced. Bars equal 1 cm. **(E)** The number of pycnidia counted from the fungal strains described in **(D)**. The data are the means ± SD (*n* = 3). The different letters indicate a significant difference at *p* < 0.01 (one-way ANOVA).

To investigate whether our *V. mali* strain can host mycoviruses and become hypovirulent, CHV1 and MyRV1 were introduced into *V. mali* via protoplast fusion using two protoplast fractions prepared from CHV1- or MyRV1-infected *C. parasitica* and virus-free *V. mali*. CHV1 and MyRV1 were also simultaneously introduced to *V. mali* through protoplast fusion using the mixture of protoplasts prepared from *C. parasitica* that were infected with CHV1 and MyRV1. After protoplast fusion, regeneration of *V. mali* protoplasts and transfer to new PDA plates, viral dsRNA accumulation in individual *V. mali* isolates was analyzed at 4 days after transference to a PDA plate. The results showed accumulations of 12.7-kilobase dsRNA of CHV1 or 11 dsRNA genome segments (4,127 to 732 bases) of MyRV1 in *V. mali* isolates regenerated after protoplast fusion, using corresponding CHV1- or MyRV1-infected *C. parasitica*. Moreover, simultaneous accumulation of CHV1 and MyRV1 dsRNAs was also detected in *V. mali* after protoplast fusion using the mixture of protoplasts prepared from *C. parasitica* that were infected with CHV1 and MyRV1 (Figure 1A). These results indicate that this *V. mali* strain is a compatible host for CHV1 and MyRV1. Notably, defective interfering RNAs (DI-RNAs) of the CHV1 genome were frequently observed in doubly infected fungi but not in singly infected fungi (Figure 1A). Infection of CHV1 in *C. parasitica* usually gives rise to DI-RNAs at a very high frequency (Shapira et al., 1991a) and DI-RNAs were also formed in *C. parasitica* doubly infected with CHV1 and MyRV1 (Supplementary Figure 1). Because *dcl2* gene is essential for production of DI-RNAs (Zhang and Nuss, 2008), it is thus possible that generation of CHV1 DI-RNAs during co-infection with MyRV1 in *V. mali* is due to the upregulation of *dcl2* gene expression (further supporting data and discussion are presented below).

### CHV1 and MyRV1 Infection Reduced the Growth and Virulence of *V. mali*

To examine the effect of CHV1 and MyRV1 infection on *V. mali*, singly and doubly infected fungi, as well as virus-free fungi, were cultured under the same conditions, and the colony growth and morphology were analyzed. *V. mali* strains infected with either virus showed a reduction in colony size, although the fungus colony infected with CHV1 was much smaller than that infected with MyRV1 and showed an irregular margin and less dense mycelia (Figures 1B,C). Moreover, the MyRV1-infected fungal colony showed a slight increase in brown-colored pigments compared with the CHV1-infected and virus-free *V. mali* strains. Similarly, in previous studies, MyRV1 infection in *C. parasitica* resulted in reduced growth of aerial hyphae and enhanced the production of brown pigments (Sun et al., 2006; Tanaka et al., 2011). *V. mali* co-infected with both viruses exhibited a smaller colony size than when infected with CHV1 alone (Figures 1B,C). Next, the formation of asexual fruiting bodies (pycnidia) of *V. mali* cultured on a PDA plate was investigated at a later growth stage of the fungus (Figure 1D). CHV1 infection reduced pycnidia production, whereas MyRV1 infection increased pycnidia production (Figure 1E). The *V. mali* strain infected with both viruses produced a similar number of pycnidia as the virus-free strain (Figure 1E), suggesting that MyRV1 infection compromised the negative effects of CHV1 infection on pycnidia production in *V. mali*.

**FIGURE 2:**
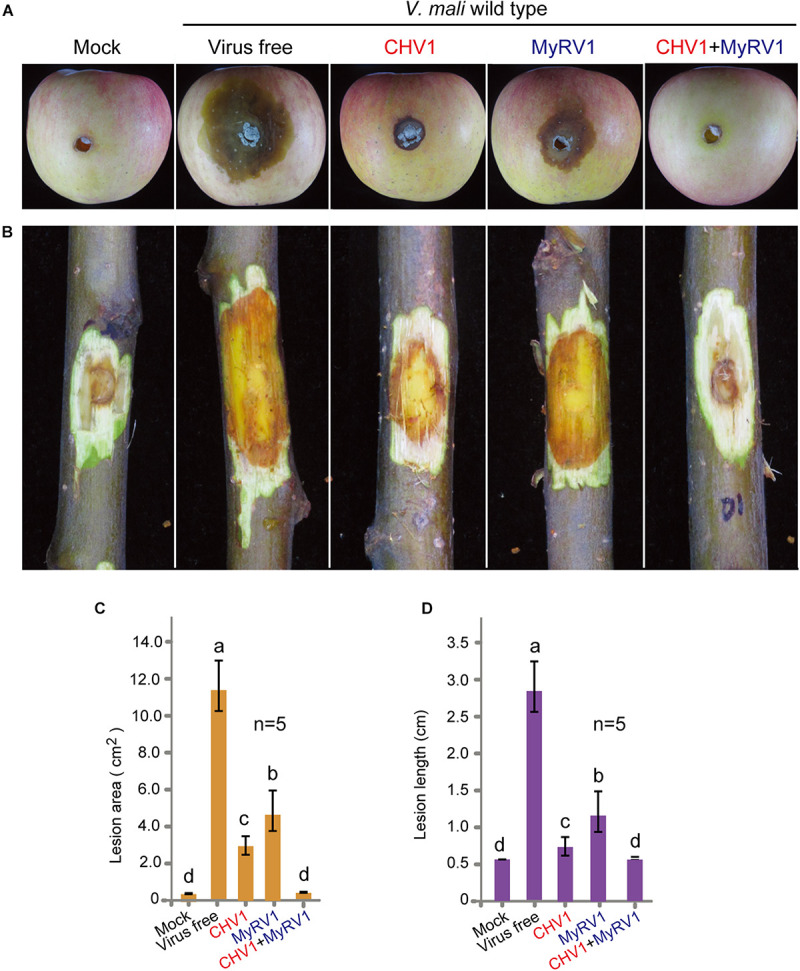
The virulence levels of *V. mali* strains singly or doubly infected with CHV1 and MyRV1. **(A)** Representative images showing lesions on apples induced by *V. mali* strains. The cankers were photographed at 5 days post-inoculation. **(B)** Representative images showing lesions on apple twigs induced by *V. mali* strains. The lesions on twigs were photographed at 5 days post-inoculation. **(C)** Lesion areas on apples measured in the experiment described in **(A)**. The data are the means ± SD (*n* = 5). The different letters indicate a significant difference at *p* < 0.01 (one-way ANOVA). **(D)** Lesion lengths on apple twigs measured in the experiment described in **(B)**. The data are the means ± SD (*n* = 5). The different letters indicate a significant difference at *p* < 0.01 (one-way ANOVA).

For the fungal virulence assay, virus-infected and virus-free *V. mali* strains (agar plugs) were inoculated in apples and the virulence levels were evaluated by measuring the area of lesions at 5 days after inoculation. The virus-free *V. mali* strain induced the largest lesions on apples, while the *V. mali* strains infected with either CHV1 or MyRV1 exhibited significantly smaller canker lesions although CHV1 infection induced a stronger effect than MyRV1 (Figures 2A,C). The doubly infected *V. mali* strain did not produce obvious lesions in apples (Figures 2A,C), suggesting that double infection strongly reduces the virulence of *V. mali*. A virulence assay was performed in parallel using apple twigs. In general, the effects of single and double infection on the virulence of *V. mali* using apple twigs observed at 5 and 10 days after inoculation were similar to those observed in apples (Figures 2B,D and Supplementary Figure 2). Our observations indicate that CHV1 and MyRV1 confer hypovirulence in *V. mali* and co-infection of these mycoviruses further reduces the virulence of the fungus. The differing levels of hypovirulence observed in this study between CHV1 and MyRV1 in *V. mali* were opposite to that observed in *C. parasitica* where MyRV1 showed a stronger effect than CHV1 in attenuating fungal virulence (Sun et al., 2006). Moreover, CHV1 and MyRV1 co-infection in *C. parasitica* did not markedly reduce the virulence of the fungal host relative to that induced by single infection (Sun et al., 2006). Thus, the molecular mechanisms underlying the hypovirulence caused by CHV1 and MyRV1 may differ between *V. mali* and *C. parasitica*.

### Co-infection With MyRV1 Causes Unstable Infection of CHV1 in *V. mali*

**FIGURE 3:**
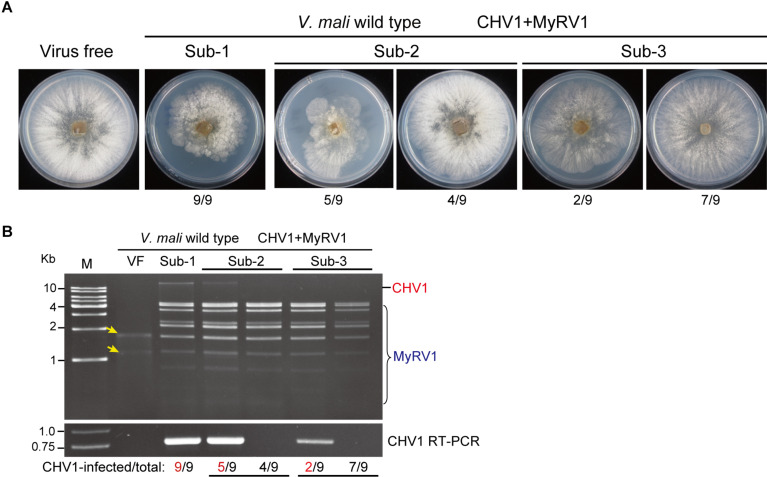
The accumulation of CHV1 and MyRV1 in co-infected *V. mali* after subsequent subculturing. **(A)** The phenotypic growth on PDA medium of representative *V. mali* strains co-infected with CHV1 and MyRV1 in the first to third fungal subcultures (Sub-1 to Sub-3). The colonies were grown on PDA for 4 days and then photographed. The numbers below the images indicate the number of fungal colonies showing a similar phenotype as presented in the photo per total number of colonies shown. **(B)** Viral dsRNA and RT-PCR analyses of doubly infected *V. mali* strains described in **(A)**. The numbers below the lanes indicate the number of samples in which CHV1 was detected or undetected per total number of samples. Yellow-colored arrows mark the traces of ribosomal RNAs in virus-free (VF) sample incorporated during dsRNA isolation.

Subsequent subculturing on PDA plates of *V. mali* strains that were co-infected with CHV1 and MyRV1 revealed that most fungal strains regained their vegetative growth rate, which contrasts with the small colony size initially exhibited by these strains (Figure 3A). dsRNA and RT-PCR analyses showed that CHV1 was undetectable or its accumulation decreased in the fungal strain that recovered to a higher growth rate, whereas MyRV1 stably accumulated in all strains after fungal subculture (Figure 3B). In the *V. mali* strains singly infected with CHV1 or MyRV1, stable virus accumulation was observed in subsequent fungal cultures (Supplementary Figure 3). These observations suggest that MyRV1 infection has an antagonistic effect on CHV1 accumulation in *V. mali*. The interactions between co-infecting viruses can be either synergistic/facilitative or antagonistic while in plant and fungi, RNA silencing mechanism has been shown to be the most common underlying mechanism of both types of virus-virus interactions (Sun et al., 2006; Syller, 2012; Chiba and Suzuki, 2015; Mascia and Gallitelli, 2016; Aulia et al., 2019; Bian et al., 2020).

### Horizontal Transmission of CHV1 and MyRV1 in *V. mali*

**FIGURE 4:**
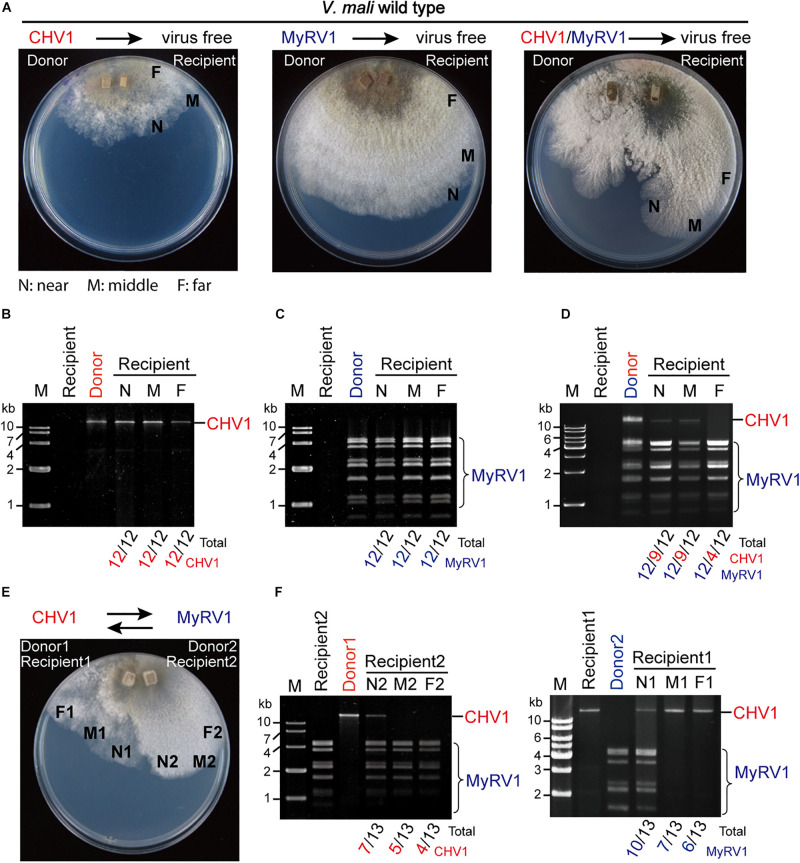
The efficiency of CHV1 and MyRV1 horizontal transmission via hyphal anastomosis in *V. mali*. **(A)** Co-culture on PDA plates of the virus-free *V. mali* strain and the virus-infected *V. mali* strain as recipient and donor viruses, respectively. After 1 week of hyphal contact, mycelial plugs were removed from three locations (a near, middle, and far distance from the hyphal fusion areas) in the recipient side, transferred onto new PDA plates and cultured in PDB for dsRNA extraction. **(B–D)** Detection of viral dsRNAs in recipient strains co-cultured with CHV1-infected **(B)**, MyRV1-infected **(C)**, and CHV1 + MyRV1-infected strains **(D)**. The numbers below the lanes indicate the number of samples in which viral dsRNAs were detected per total number of samples. **(E)** Co-culture of CHV1-infected and MyRV1-infected *V. mali* strains on a PDA plate. After 1 week of hyphal contact, mycelial plugs were removed from three locations on both sides, transferred onto new PDA plates and cultured in PDB for dsRNA extraction. **(F)** Detection of viral dsRNAs in fungal strains obtained in the co-culture experiment described in **(E)**.

To examine whether CHV1 and MyRV1 were horizontally transmitted in *V. mali* via hyphal anastomosis, a virus-infected strain (donor), and a virus-free strain (recipient) were co-cultured on a PDA plate and after 5 days of hyphal fusion, agar plugs taken from the recipient colony side were grown in PDB culture and subjected to dsRNA extraction. As no specific marker gene or antibiotic resistance is available in the recipient strains for selection, the mycelial plugs, which were taken from recipient side may include the donor cells. To discern such possibility, agar plugs were taken from three positions that are near, middle, and far distance from the hyphal fusion areas in the recipient colony. Following hyphal fusion on the PDA plate, the virus-free *V. mali* strain co-cultured with the CHV1-infected strain showed a CHV1-infected phenotype, while the virus-free strain co-cultured with the MyRV1-infected strain showed no obvious phenotypic change, due to a slightly altered phenotype of MyRV1-infected strain on the PDA plate (Figure 4A). dsRNA analysis showed the accumulation of CHV1 or MyRV1 dsRNAs in all fungal strains taken from the three different positions in the recipient colonies (Figures 4B,C). Intriguingly, in hyphal fusion with the CHV1 and MyRV1 co-infected strain, the virus-free strain did not showed altered phenotype as observed for hyphal fusion using the CHV1-infected strain (Figure 4A). Accordingly, the fungal strains taken from the recipient colonies co-cultured with the CHV1 and MyRV1 co-infected strain as a donor, all accumulated MyRV1 dsRNAs, however, several strains accumulated no or low levels of CHV1 dsRNA, in particular to a higher number when mycelial plugs were taken far from the hyphal fusion areas (Figure 4D). Although in this assay we cannot rule out the possibility of the presence of donor strains in some of fungal strains taken from the recipient colonies, the results of our co-culture assays using up to 12 PDA plates for each single and double infection suggest that co-infection with MyRV1 interferes with the efficient horizontal transmission of CHV1 in *V. mali*. To further examine whether the co-infection condition affects the horizontal transmission of CHV1 and MyRV1, CHV1- and MyRV1-infected strains were co-cultured on 13 PDA plates to allow for hyphal anastomosis (Figure 4E). dsRNA analysis showed that neither CHV1 nor MyRV1 were efficiently transmitted to the recipient side of the fungus (Figure 4F), suggesting that CHV1 and MyRV1 have an antagonistic effect on each other concerning horizontal transmission.

Mycoviruses are transmitted vertically from the mycelium to spores or horizontally via hyphal anastomosis (Hillman et al., 2018). Therefore, transmission efficiency is an important factor when evaluating a mycovirus for its potential as a biological control agent. Under natural conditions, the spreading of mycoviruses is limited by vegetative incompatibility among fungal species and strains in a fungal population (Choi et al., 2012), however, some studies on filamentous phytopathogenic fungi have demonstrated the occurrence of virus transmission between vegetatively incompatible fungal hosts (Brusini and Robin, 2013; Hamid et al., 2018). Sclerotinia sclerotiorum Mycoreovirus 4 (genus *Mycoreovirus* of the family Reoviridae), which originated from the phytopathogenic fungus *Sclerotinia sclerotiorum*, can suppress host non-self recognition and thus enable the horizontal transmission of heterologous viruses (Wu et al., 2017). Our research group recently proposed a model in which mycoviruses could spread across vegetatively incompatible fungal strains or to different fungal species through plant–fungal-mediated routes facilitated by plant viruses (Bian et al., 2020). Conversely, the present study showed that the efficiency of CHV1 and MyRV1 horizontal transmission is reduced under co-infection conditions. Further investigations should be conducted to assess whether such inhibitory effects on horizontal transmission between co-infected viruses commonly occurs among mycoviruses.

### CHV1 and MyRV1 Infection Up-regulates the Transcript Expression of RNA Silencing-Related Genes in *V. mali*

**FIGURE 5:**
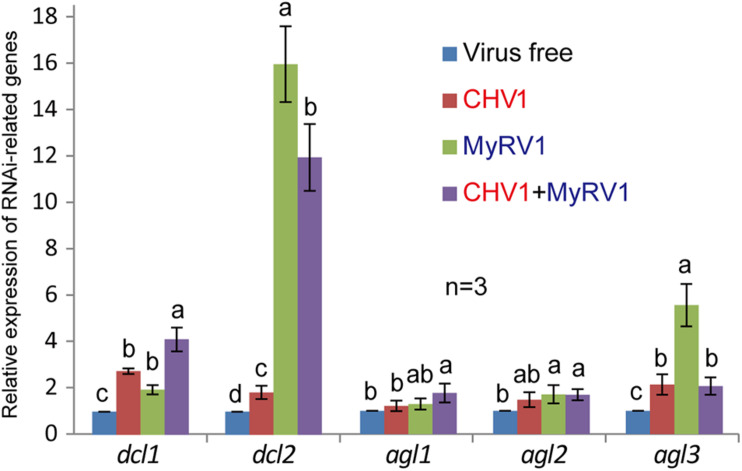
The relative transcript expression of RNA silencing-related genes in *V. mali* strains singly or doubly infected with CHV1 and MyRV1. The transcript expression of *V. mali dcl1*, *dcl2*, *agl1*, *agl2*, and *agl3* genes following single and double virus infection were analyzed by quantitative RT-PCR. The data are the means ± SD (*n* = 3). The different letters indicate a significant difference at *p* < 0.01 (one-way ANOVA).

CHV1 and MyRV1 infection in *C. parasitica* was shown to increase the transcript expression of *dcl2* and *agl2* genes, two key components of the antiviral RNA silencing pathway in *C. parasitica* (Sun et al., 2009; Chiba and Suzuki, 2015; Andika et al., 2017). To examine whether such effects of CHV1 and MyRV1 infection also occur in *V. mali*, transcript expression of *V. mali dcl1*, *dcl2*, *agl1*, *agl2*, and *agl3* genes were analyzed by quantitative RT-PCR following single and double virus infection. The analyses revealed that single infection and co-infection generally enhanced the transcript expression of all the genes, however, the highest increase in expression was observed for *dcl2* and *agl3*, particularly following MyRV1 infection (Figure 5). This suggests that RNA silencing is activated by CHV1 or MyRV1 infection in *V. mali*, however, CHV1 may suppress the activation of the RNA silencing response through the activity of the CHV1-encoded p29 silencing suppressor, as previously demonstrated in *C. parasitica* (Sun et al., 2009; Chiba and Suzuki, 2015). Moreover, much more increase of *dcl2* transcript levels following co-infection of CHV1 and MyRV1 than CHV1 single infection supports the notion that generation of CHV1 DI-RNAs during co-infection with MyRV1 (Figure 1A) is related to the high expression of *dcl2* gene.

### The Inactivation of *dcl2* in *V. mali* Enhances the dsRNA Accumulation and Stability of CHV1

**FIGURE 6:**
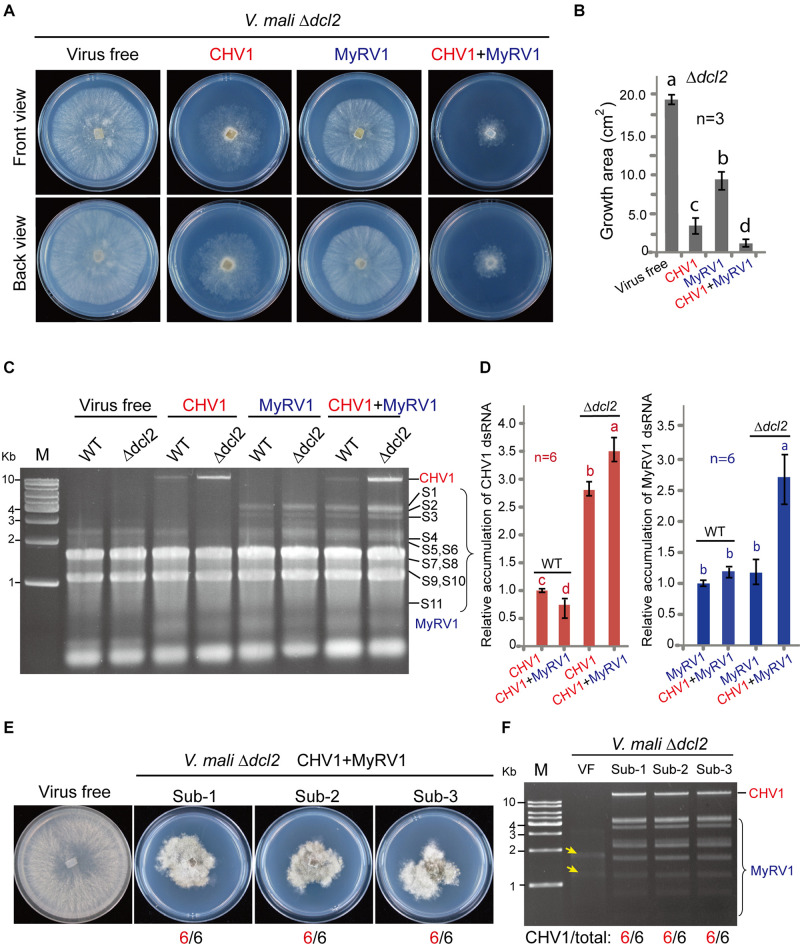
The accumulation of CHV1 and MyRV1 in the *dcl2* knockout mutant of *V. mali*. **(A)** The phenotypic growth on PDA medium of the representative *V. mali* mutant strains singly or doubly infected with CHV1 and MyRV1. The colonies were grown on PDA for 4 days and then photographed. **(B)** The growth areas of the fungal strains described in **(A)**. The data are the means ± SD (*n* = 3). The different letters indicate a significant difference at *p* < 0.01 (one-way ANOVA). **(C)** Agarose gel electrophoresis of the total RNA samples extracted from the wild-type and *dcl2* knockout mutant of *V. mali* strains singly or doubly infected with CHV1 and MyRV1. The total RNA samples were analyzed via electrophoresis in agarose gel stained with ethidium bromide. **(D)** The relative dsRNA accumulation levels of CHV1 and MyRV1 in the wild-type and Δ*dcl2* knockout mutant. The dsRNA bands detected in the experiment described in **(C)** were quantified and analyzed using Image J Macro software. The data are the means ± SD (*n* = 6). The different letters indicate a significant difference at *p* < 0.01 (one-way ANOVA). **(E)** The phenotypic growth on PDA medium of the representative *V. mali* mutant strains co-infected with CHV1 and MyRV1 in the first to third fungal subcultures (Sub-1 to Sub-3). The colonies were grown on PDA for 6 days and then photographed. The numbers below the images indicate the number of fungal colonies showing a similar phenotype as that presented in the photo per total number of colonies. **(F)** Viral dsRNA analysis of the doubly infected *V. mali* mutant strains described in **(E)**. The numbers below the lanes indicate the number of samples in which CHV1 dsRNAs were detected per total number of samples. Yellow-colored arrows mark the traces of ribosomal RNAs in virus-free (VF) sample incorporated during dsRNA isolation.

To investigate whether RNA silencing contributes to the inhibition of mycovirus multiplication in *V. mali*, CHV1 and MyRV1 were introduced to a *dcl2* knockout mutant (Δ*dcl2*) of *V. mali* by hyphal anastomosis. Single infection or co-infection of CHV1 and MyRV1 reduced Δ*dcl2* growth and produced a similar pattern as that observed in the *V. mali* wild-type (Figures 6A,B). Next, the relative viral dsRNA accumulation levels in the wild-type and Δ*dcl2* mutant were compared by extracting the total RNA from the infected fungi. The accumulation of the viral dsRNAs was visualized through agarose gel electrophoresis. CHV1 dsRNA accumulation was enhanced in the Δ*dcl2* mutant in the case of single infection or co-infection with MyRV1, while MyRV1 dsRNA accumulation levels were similar in the wild-type and Δ*dcl2* mutant during single infection but were enhanced in the Δ*dcl2* mutant during co-infection with CHV1 (Figures 6C,D). This suggests that MyRV1 is less affected by antiviral RNA silencing responses in *V. mali* than CHV1. This is similar to an observation in *C. parasitica* where the inactivation of *dcl2* increased CHV1 RNA accumulation but had no effect on MyRV1 RNA accumulation (Chiba and Suzuki, 2015). It was also observed that co-infection reduced CHV1 dsRNA accumulation relative to that of single infection but had no effect on MyRV1 dsRNA accumulation (Figures 6C,D). The Δ*dcl2* mutant strains co-infected with CHV1 and MyRV1 maintained restrained vegetative growth in successive fungal subcultures (Figure 6E). Accordingly, CHV1 stably accumulated after successive fungal subculturing in the Δ*dcl2* mutant co-infected with these two viruses (Figure 6F), similar as the stability of CHV1 and MyRV1 single infection in the Δ*dcl2* mutant (Supplementary Figure 3). These results indicate that DCL2 is responsible for the suppression of CHV1 accumulation during co-infection with MyRV1. Notably, formation of CHV1 DI-RNAs was not observed in co-infected Δ*dcl2* mutant (Figure 6F), further supporting the view that a high expression of *dcl2* transcripts during co-infection with MyRV1 is responsible for generation of CHV1 DI-RNAs.

Previous studies have shown that DCL2 (but not DCL1) is critical for antiviral RNA silencing in *C. parasitica* and *Colletotrichum higginsianum* (Segers et al., 2007; Campo et al., 2016), while DCL2 and DCL1 are functionally redundant in antiviral RNA silencing in *Fusarium graminearum*, *S. sclerotiorum*, and *Neurospora crassa* (Yu et al., 2018; Neupane et al., 2019; Honda et al., 2020). To a lesser degree, CHV1 or MyRV1 infection also increased the expression of *dcl1* transcripts (Figure 5). Further research is required using single and double *dcl* mutants to assess whether DCL1 also contributes to antiviral RNA silencing in *V. mali*.

### The Inactivation of *dcl2* Restores the Horizontal Transmission Efficiency of CHV1 and MyRV1 During Co-infection

**FIGURE 7:**
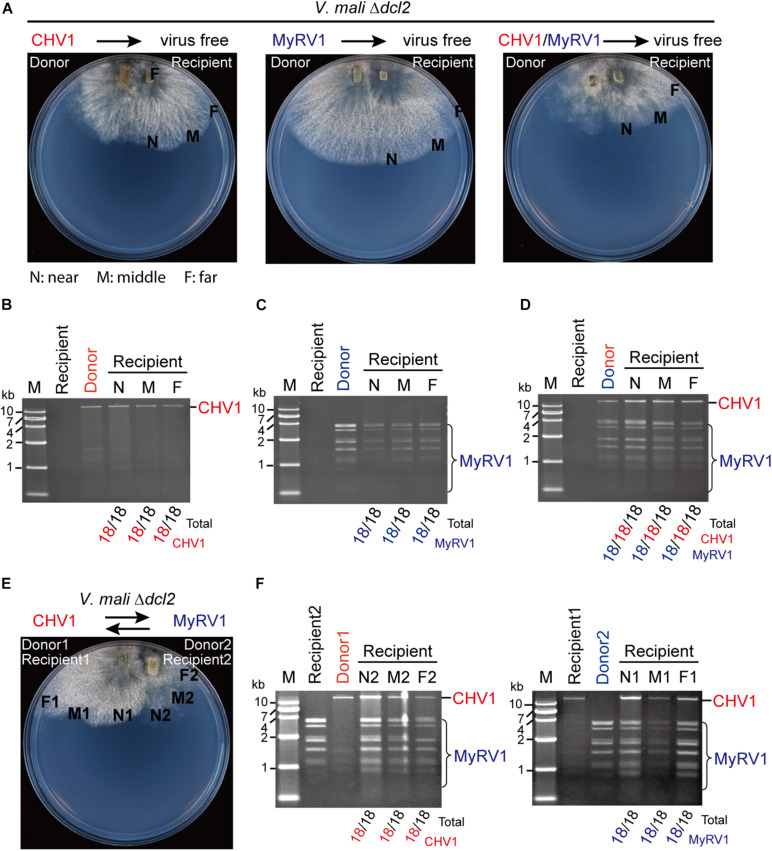
The efficiency of CHV1 and MyRV1 horizontal transmission via hyphal anastomosis in the *dcl2* knockout mutant of *V. mali*. **(A)** Co-culture on PDA plates of the virus-free *V. mali* mutant strain and the virus-infected *V. mali* mutant strain as recipient and donor viruses, respectively. After 1 week of hyphal contact, mycelial plugs were taken from three locations (a near, middle, and far distance from the hyphal fusion areas) in the recipient side, transferred onto new PDA plates and cultured in PDB for dsRNA extraction. **(B–D)** Detection of viral dsRNAs in recipient strains co-cultured with CHV1-infected **(B)**, MyRV1-infected **(C)**, and CHV1 + MyRV1-infected strains **(D)**. The numbers below the lanes indicate the number of samples in which viral dsRNAs were detected per total number of samples. **(E)** Co-culture on a PDA plate of CHV1-infected and MyRV1-infected *V. mali* mutant strains. After 1 week of hyphal contact, mycelial plugs were removed from three locations on both sides, transferred onto new PDA plates and cultured in PDB for dsRNA extraction. **(F)** Detection of viral dsRNAs in fungal strains obtained in the co-culture experiment described in **(E)**.

The effect of *dcl2* inactivation on the horizontal transmission of CHV1 and MyRV1 through hyphal fusion was also examined by co-culturing on 18 PDA plates for each donor and recipient combination. CHV1 and MyRV1 were efficiently transmitted to the virus-free Δ*dcl2* mutant under single or double infection conditions (Figures 7A–D). Moreover, when CHV1- and MyRV1-infected Δ*dcl2* mutants were co-cultured on a PDA plate, allowing for hyphal fusion (Figure 7E), both viruses were efficiently transmitted to the recipient side of the fungus (Figure 7F). These observations suggest that the efficiency of transmission of CHV1 is restored by the fact that its accumulation is increased in the Δ*dcl2* mutant and indirectly, the higher concentration of CHV1 could also enhance the transference of MyRV1 transmission.

Multiple infections of mycoviruses in a single host are common in nature and, therefore, interplays between or among mycoviruses may occur (Thapa and Roossinck, 2019). Several studies on virus–virus interaction in *C. parasitica* demonstrated that an RNA silencing mechanism is implicated in either synergistic or antagonistic effects between viruses. In the case of a one-way interaction between CHV1 and MyRV1, CHV1 enhances MyRV1 replication and induces frequent generation of MyRV1 rearrangements (Sun et al., 2006; Sun and Suzuki, 2008). The p29 RNA silencing suppressor encoded by CHV1 plays an important role in synergism and MyRV1 rearrangement (Sun and Suzuki, 2008; Tanaka et al., 2011). A study on the interactions between two other members of genus *Hypovirus* and *Mycoreovirus* showed that Cryphonectria hypovirus 4 facilitates the stable infection and enhanced vertical transmission of Mycoreovirus 2, through suppressing the induction of *dcl2* transcripts (Aulia et al., 2019). Chiba and Suzuki (2015) demonstrated that the induction of *dcl2* transcripts by co-infection with MyRV1, or a mutant CHV1 lacking the p29 RNA silencing suppressor, leads to suppression of Rosellinia necatrix victorivirus 1 (genus *Totivirus* of family *Totiviridae*) multiplication, indicating an RNA silencing-mediated one-way interference between unrelated viruses. Similarly, the results of the present study demonstrated that co-infection with MyRV1 compromised the stability of infection and horizontal transmission of CHV1 in *V. mali* (Figures 3, 4) and this was associated with highly up-regulated *dcl2* transcripts (Figure 5). Moreover, the inactivation of *dcl2* restored the stability and horizontal transmission efficiency of CHV1 in the presence of MyRV1 (Figure 6). This suggests that MyRV1 infection has inhibitory effects on CHV1 multiplication and horizontal transmission in *V. mali* through the activation of antiviral RNA silencing.

## Conclusion

*Valsa mali* causes Valsa canker which penetrates deeply into the phloem and xylem of apple tree; therefore, the disease cannot be controlled effectively through chemical treatments. Research interest in mycoviruses has increased due to their potential use as a biological control agent for crop fungal diseases. Thus, expansion of the host range of hypovirulent mycoviruses and studies on virus-fungal host interactions could facilitate the development of biological control measures for fungal diseases. Hence, we introduced two hypovirulent mycoviruses (CHV1 and MyRV1) which originated from *C. parasitica* to *V. mali* using protoplast fusion. Overall, the results of this study revealed that infection with CHV1 and, to a lesser extent, MyRV1 markedly reduced the vegetative growth and virulence of *V. mali*. Therefore, these mycoviruses are potential biological control agents for Valsa canker disease. Moreover, the results demonstrated that co-infection of CHV1 and MyRV1 conferred stronger hypovirulent effects on *V. mali*. However, co-infection also suppressed the infection stability and horizontal transmission efficiency of CHV1, which is disadvantageous for its application as a biological control method. The results of this study provide a scientific base for future research on the development of the practical application of mycoviruses as a biological control agent for apple Valsa canker disease in the field.

## Data Availability Statement

The raw data supporting the conclusions of this article will be made available by the authors, without undue reservation.

## Author Contributions

LSu designed the experiments. SY, RD, LSa, LH, and JL performed the experimental work. IA and LSu analyzed the data and wrote the manuscript. All authors contributed to the article and approved the submitted version.

## Conflict of Interest

The authors declare that the research was conducted in the absence of any commercial or financial relationships that could be construed as a potential conflict of interest.

## Supplementary Material

Supplementary Figure 1 Agarose gel electrophoresis of dsRNAs extracted from *C. parasitica* strains doubly infected with CHV1 and MyRV1.

Supplementary Figure 2 **(A)** Representative images showing lesions on apple twigs induced by *V. mali* strains. The lesions on twigs were photographed at 10 days post-inoculation. **(B)** Lesion areas on apples measured in the experiment described in **(A)**. The data are the means ± SD (*n* = 5). The different letters indicate a significant difference at *p* < 0.01 (one-way ANOVA).

Supplementary Figure 3 Agarose gel electrophoresis of dsRNAs extracted from wild-type and Δ*dcl2* mutant of *V. mali* strains singly infected with CHV1 or MyRV1. The numbers below the lanes indicate the number of samples in which viral dsRNAs were detected per total number of samples.

Supplementary Table 1 List of primers used in this study.
