# Supplementary figures and images for: Infection of Two Heterologous Mycoviruses Reduces the Virulence of Valsa mali, a Fungal Agent of Apple Valsa Canker Disease
Source: Front Microbiol. 2021 May 25;12:659210. doi: 10.3389/fmicb.2021.659210 (PMC8186502; doi:10.3389/fmicb.2021.659210)

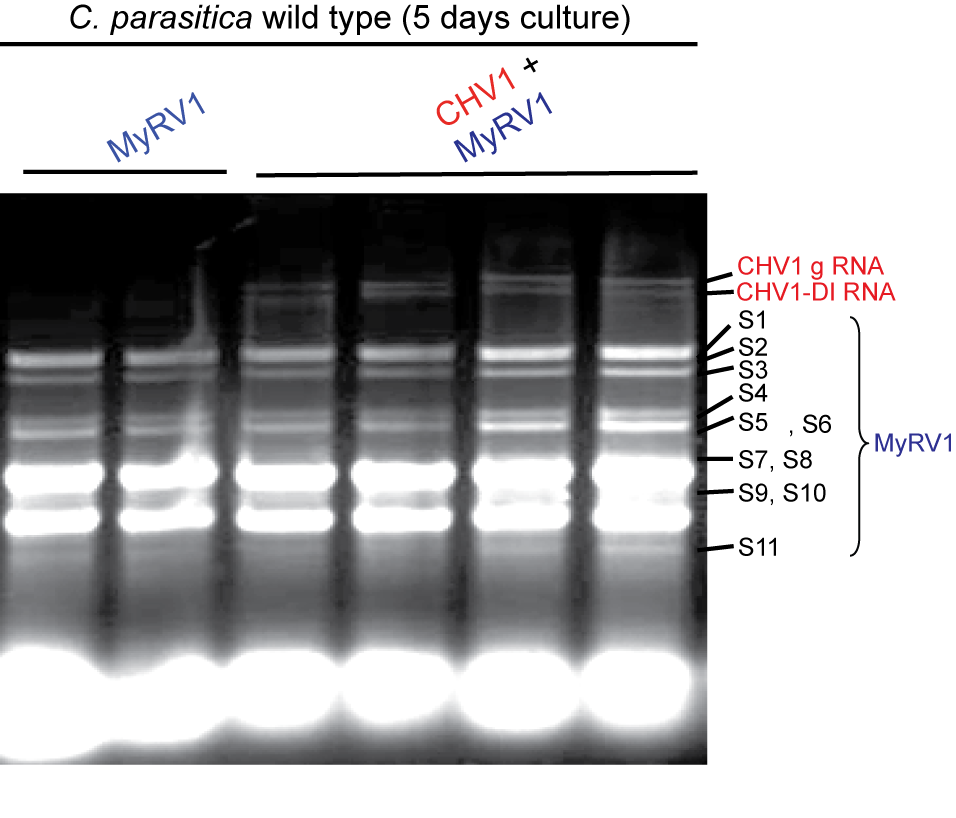

Supplement: Supplementary Figure 1 — Agarose gel electrophoresis of dsRNAs extracted from C. parasitica strains doubly infected with CHV1 and MyRV1. [file Image_1.tif]

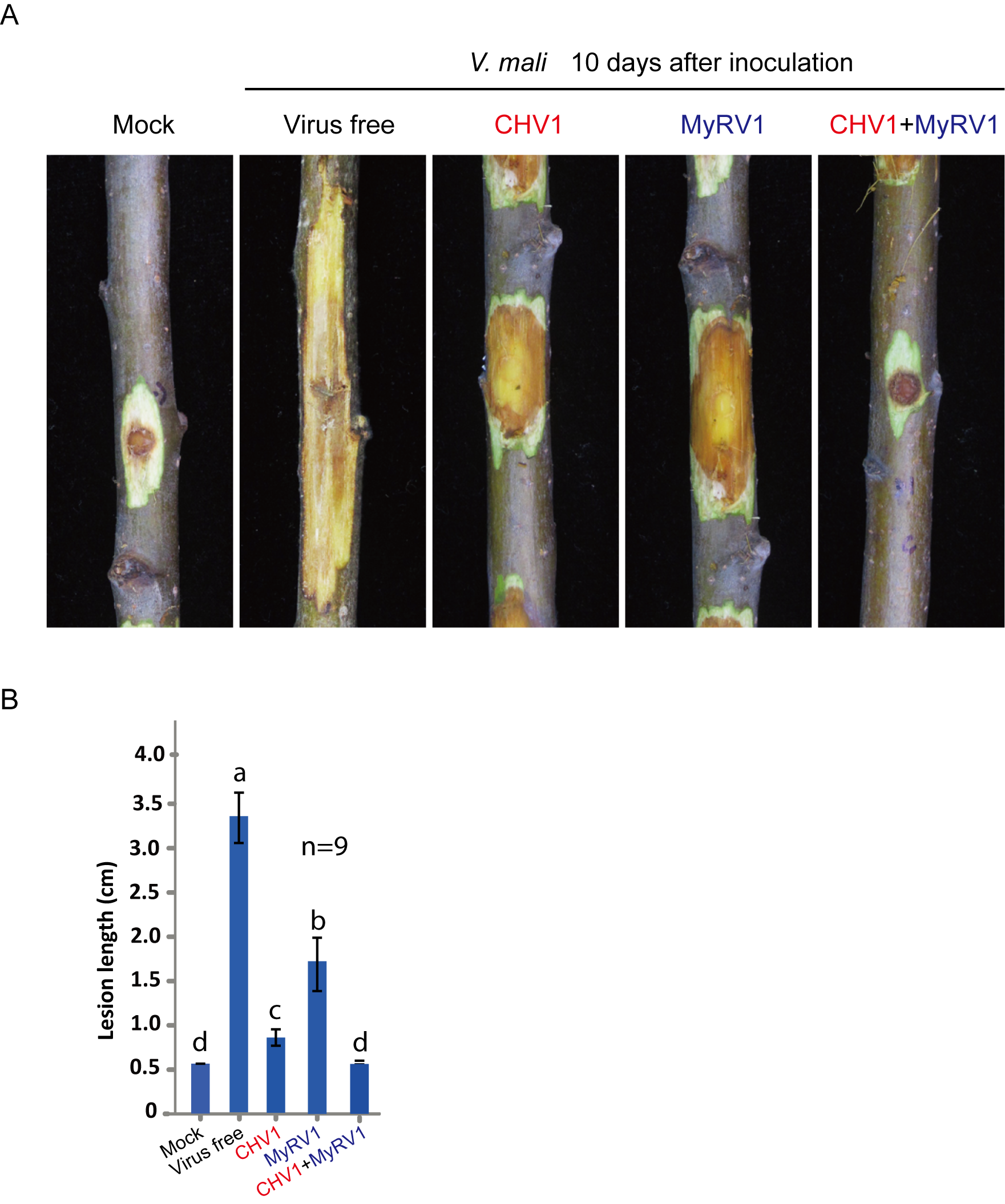

Supplement: Supplementary Figure 2 — (A) Representative images showing lesions on apple twigs induced by V. mali strains. The lesions on twigs were photographed at 10 days post-inoculation. (B) Lesion areas on apples measured in the experiment described in (A). The data are the means ± SD (n = 5). The different letters indicate a significant difference at p < 0.01 (one-way ANOVA). [file Image_2.TIF]

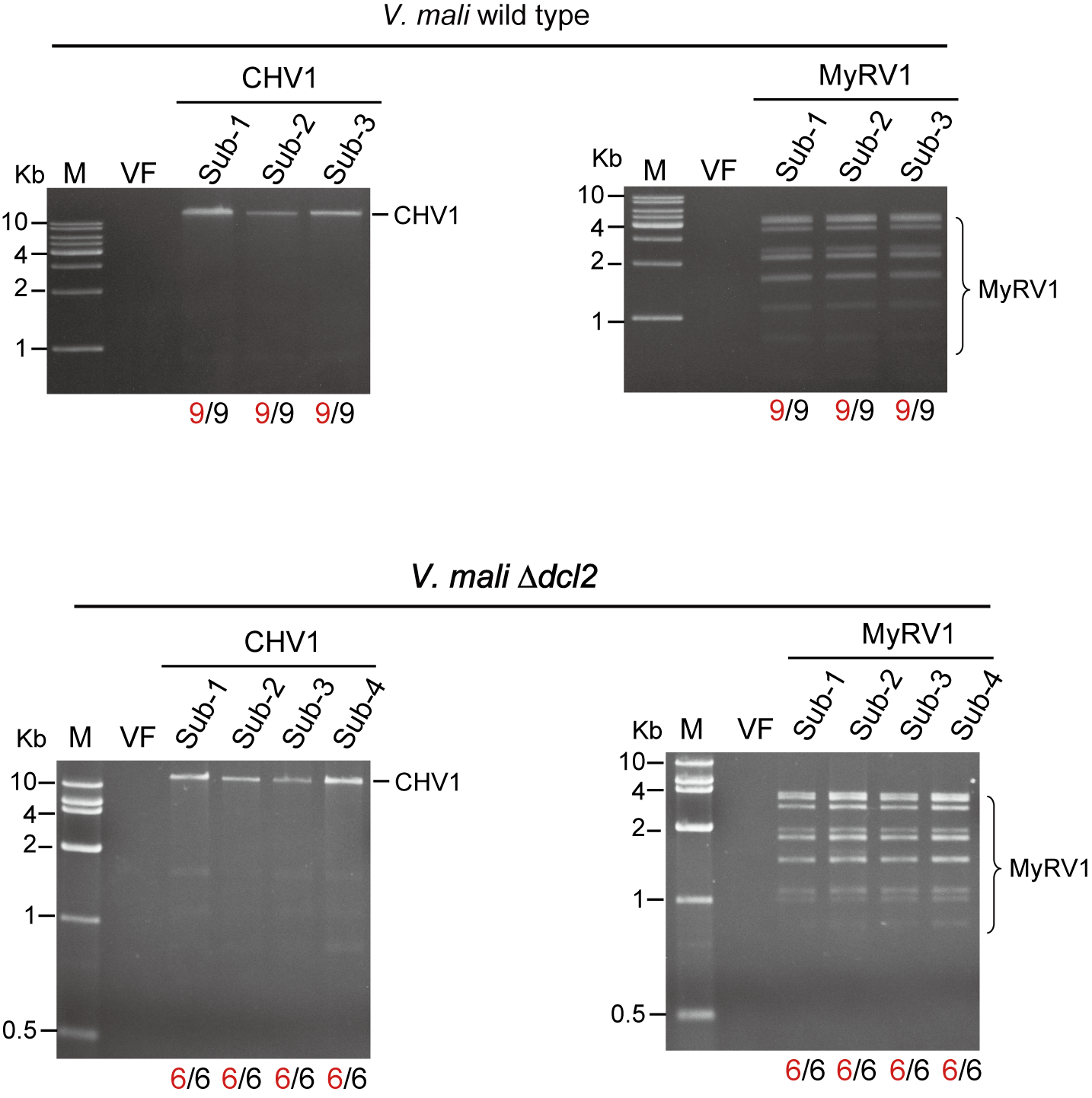

Supplement: Supplementary Figure 3 — Agarose gel electrophoresis of dsRNAs extracted from wild-type and Δdcl2 mutant of V. mali strains singly infected with CHV1 or MyRV1. The numbers below the lanes indicate the number of samples in which viral dsRNAs were detected per total number of samples. [file Image_3.TIF]
