# Supplementary material for: Infection of Two Heterologous Mycoviruses Reduces the Virulence of Valsa mali, a Fungal Agent of Apple Valsa Canker Disease
Source: Front Microbiol. 2021 May 25;12:659210. doi: 10.3389/fmicb.2021.659210 (PMC8186502; doi:10.3389/fmicb.2021.659210)
Supplement: Supplementary Table 1 — List of primers used in this study. [file Table_1.DOCX]

**Table S1.** A list of primers used in this study.

| Primer Name | Oligonucleotide sequence (5’- 3’) | GenBank Accession Number | Size of PCR product |
| --- | --- | --- | --- |
| CHV1-P29F | ATGGCTCAATTAAGAAAACCCAGTC | M57938.1 |  |
| CHV1-P29R | TCGGCCGCCAATCCGGGCAAG | M57938.1 | 775 bp |
| ITS4 | TCCTCCGCTTATTGATATGC | - |  |
| ITS5 | GGAAGGTAAAAGTCAAGG | - | 550bp |
| VmDCL2-1F | ATGGCATACTATAGTGACTCCTCCG | KUI73784.1 |  |
| VmDCL2-270R | TGATCCTGAGAACGGCAACTTG | KUI73784.1 | 270bp |
| VmDCL1-1F | ATGCCCAGAATGCATCAAGGTG | KUI68257.1 |  |
| VmDCL1-221R | TGGACTAGCCTGCTCTCCTCTTC | KUI68257.1 | 221 bp |
| VmAgo1-2426F | ATGAGGTCGAGGAGGTGCGCAAG | KT191024.1 |  |
| VmAgo1-2695 | TGCCAACTTCGTCGTGGAGGATG | KT191024.1 | 270 bp |
| VmAgo2-1F | ATGTCGGGTCACGGTGGTCATC | KT191022.1 |  |
| VmAgo2-202R | TGCCTTGAGTTCCAAAGCCAGG | KT191022.1 | 202bp |
| VmAgo5-2378F | ATCGATGTCACTCACCCCTCACC | KT191023.1 |  |
| VmAgo5-2565R | TCCTAAGGTCTTCCAGTTGCC | KT191023.1 | 188bp |
